# Larval cryopreservation as new management tool for threatened clam fisheries

**DOI:** 10.1038/s41598-021-94197-2

**Published:** 2021-07-29

**Authors:** P. Heres, J. Troncoso, E. Paredes

**Affiliations:** Centro de Investigación Mariña, Departamento de Ecoloxía e Bioloxía Animal, Laboratorio de Ecoloxía Costeira (ECOCOST), Universidade de Vigo, Vigo, Spain

**Keywords:** Developmental biology, Ecology, Biodiversity, Conservation biology

## Abstract

Cryopreservation is the only reliable method for long-term storage of biological material that guarantees genetic stability. This technique can be extremely useful for the conservation of endangered species and restock natural populations for declining species. Many factors have negatively affected the populations of high economical value shellfish in Spain and, as a result, many are declining or threatened nowadays. This study was focused on early-life stages of *Venerupis corrugata*, *Ruditapes decussatus* and *Ruditapes philippinarum* to develop successful protocols to enhance the conservation effort and sustainable shellfishery resources. Firstly, common cryoprotecting agents (CPAs) were tested to select the suitable permeable CPA attending to toxicity. Cryopreservation success using different combinations of CPA solutions, increasing equilibrium times and larval stages was evaluated attending to survival and shell growth at 2 days post-thawing. Older clam development stages were more tolerant to CPA toxicity, being ethylene-glycol (EG) and Propylene-glycol (PG) the least toxic CPAs. CPA solution containing EG yielded the highest post-thawing survival rate and the increase of equilibration time was not beneficial for clam larvae. Cryopreservation of trochophores yielded around 50% survivorship, whereas over 80% of cryopreserved D-larvae were able to recover after thawing.

## Introduction

**Figure 1:**
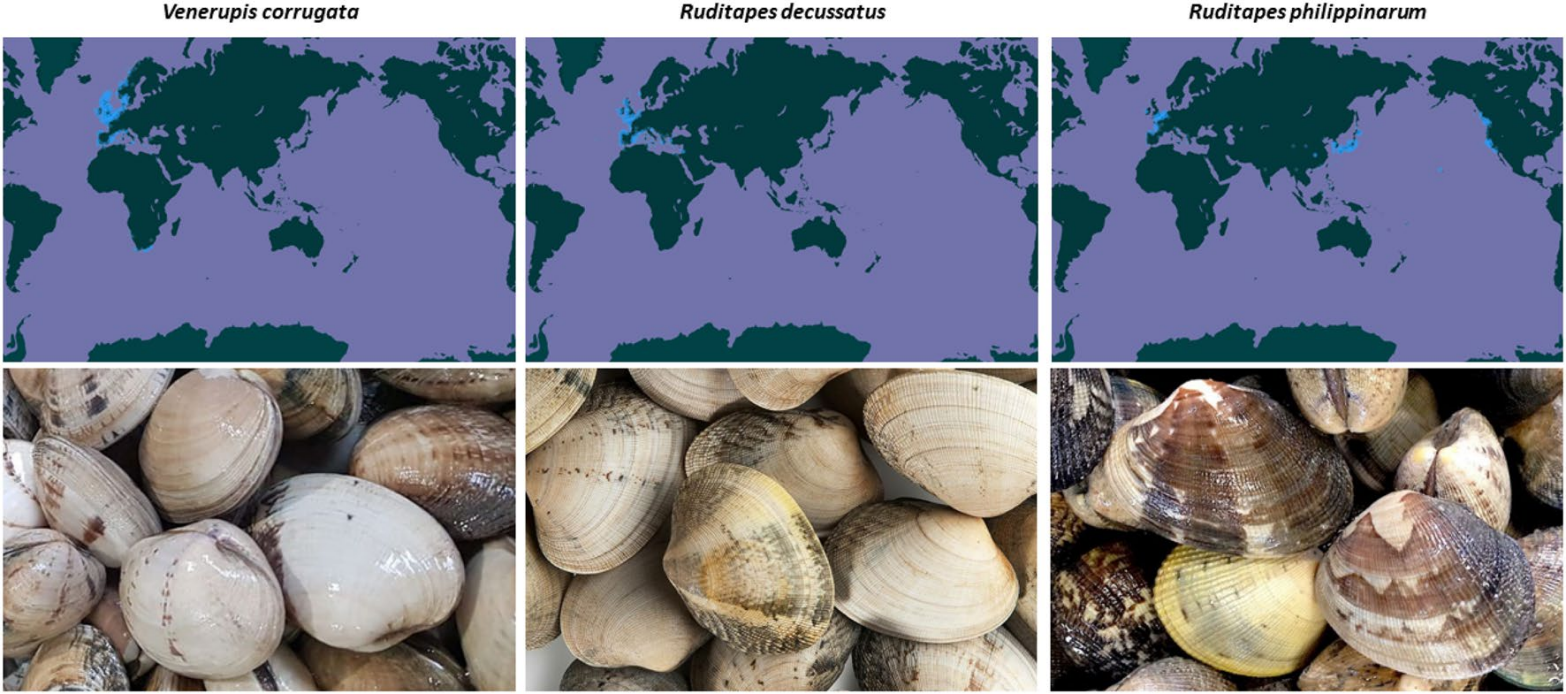
Georeferenced records of *Venerupis corrugata*, *Ruditapes decussatus* and *Ruditapes philippinarum* from 1800 to 2021. Source: GBIF.es.

The Japanese carpet shell *(Ruditapes philippinarum* (Adams & Reeve, 1850)), the grooved carpet shell (*Ruditapes decussatus* (Linnaeus, 1758)) and the slug carpet shell (*Venerupis corrugata* (Gmelin, 1971)) belong to phylum Mollusca, class Bivalvia, which comprises around 20,000 aquatic and marine species. These clams are laterally comprised with two lateral valves. Their shell is solid, equivalve, inequilateral and oval in outline whose degree can vary among species and individuals. The shell is comprised of concentric and radial lines, which are marked in *Ruditapes* genus and more pronounced in the grooved carpet shell than in the Japanese carpet shell. In the slug carpet shell, the colour of the inner layer is white, changing to deep blue along posterior edge. Externally, the colour ranges from white to increasing brownish tones. It can measure over 51 mm (Fig. 1). In the case of the grooved carpet shell, inner layers are glossy white, often with yellow or orange tints with a bluish tinge along dorsal edge. Externally, the colour palette ranges from cream to light brown, often with darker markings and measures an average of 76 mm (Fig. 1). The inner layer of the Japanese carpet shell is white with orange tints, sometimes with purple over a wide area below the umbones. The external surface is extremely variable in colour and pattern, from polished white, yellow or light brown; sometimes with stripes, steaks, blotches or zigzags of a darker brown, slightly polished (Fig. 1). The shell of this species is of 57 mm^1–4^.

**Figure 2:**
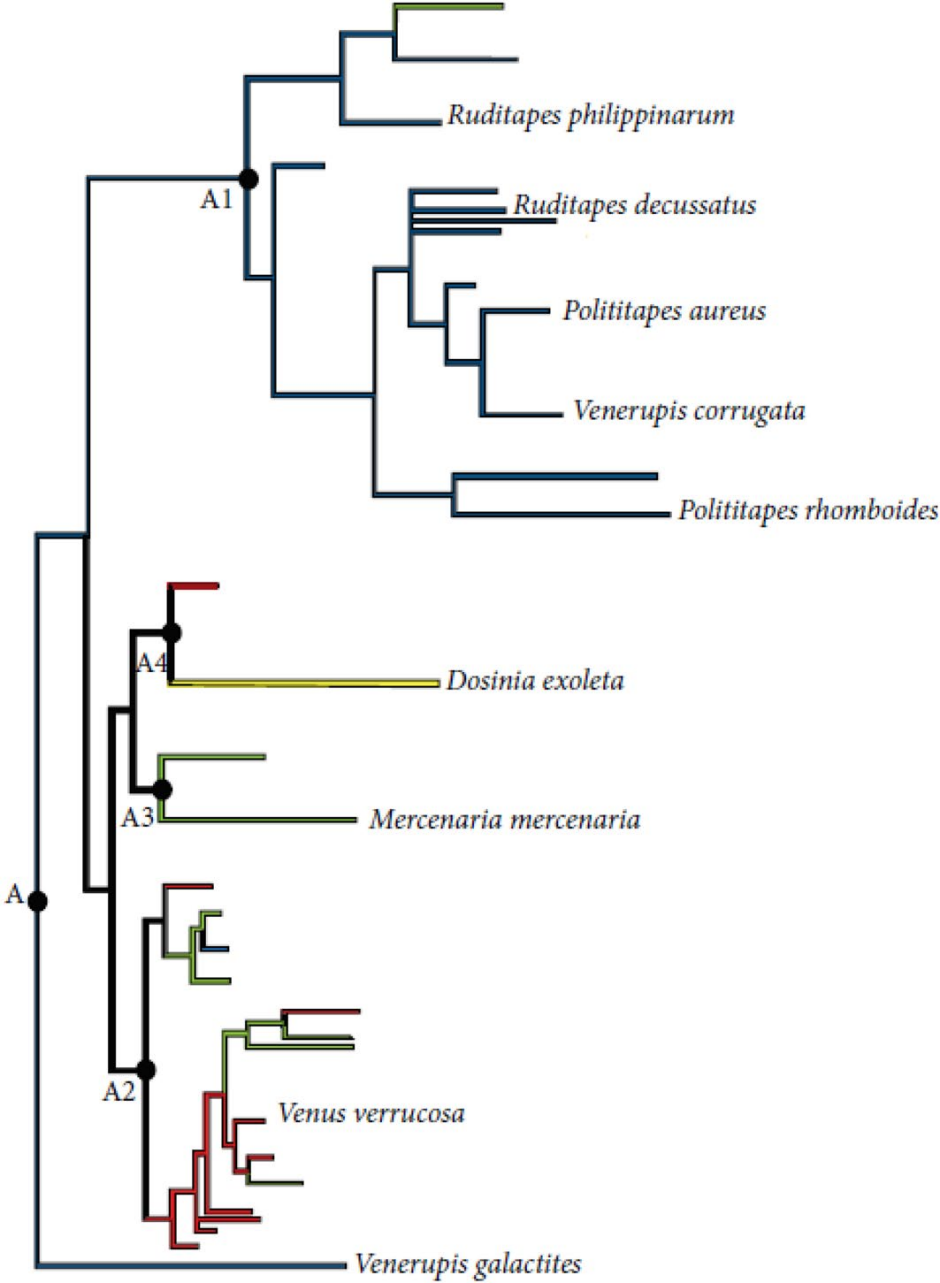
Phylogenetic tree proposed by^93^. Modified from^5^.

Most recent phylogenetic and rDNA analysis suggest that these species are close performing a cluster, which has been named traditionally Venerinae (subfamily)^1–3,5^ (Fig. 2). Adults are provided by 2 siphons and filtrate water to catch phytoplankton as food. The attachment of both siphons can be considered as a character for diagnosis: *V. corrugata* has both siphons attached along their whole length, while in *R. philippinarum* are partially bonded and completely separated in *R. decussatus*^1–3^. Most of individuals have separated sexes although hermaphrodites have been found infrequently. Eggs are released into the sea water (from 1 to 12 × 10^6^ eggs per female) and are fertilized externally. Under optimal in vitro conditions, fertilized eggs develop into trochophore larvae after 18–20 h post-fertilization (18–20 °C, constant aeration and 20–40 individuals/mL), which can swim due to the development of cilia. Approximately 24 h later, trochophores metamorphoses into D-larvae provided by a prodisoconch I, secreted by the shell gland and mantle epithelium with D-shape (90 µm). The secretion of a second larval shell (prodisoconch II) begins immediately inside pordisoconch I. At this stage, larvae can swim and feed. After the natatorial larval phase covered by 10–15 days, larvae average from 235 to 500 µm and develop an eye spot and pedal organ whereby they are able to settle. This stage is called pediveliger larva. After settlement, larvae secret byssal threads and metamorphosis involving reorientation of structures, increasing tissue complexity and secretion of adult shell^1–3,6–14^ (Joaquim et al. 2016).

The three species have a similar distribution (Fig. 1): they are extended across European Atlantic coast, from Norway to Senegal. They are also present in the Mediterranean basin. One exception is *R. philippinarum*, which is originate from Japan and extended to Southeastern Asia. It has been introduced in Hawaiian Islands, West coast of Northern America and Europe by invasions, accidental procedures (release of ballast water or sea water systems) and deliberate translocations for aquaculture purposes^1–4,15^. In natural environment, the adults live buried in sand, gravel or mud bottoms from low-tide level or intertidal zone, from a few cm to certain meters^1–4^.

These clams are between the most farmed and produced mollusks in the European Union. In 2018, over 667,934 tonnes of aquaculture mollusks were harvested in the European Union with a first sale value of 1,107 million Euros, of which the Japanese carpet shell represented 4.95% (165.4 million Euros), the grooved carpet shell 0.33% (28.4 million Euros) and the slug carpet shell 0.04% (2.9 million Euros). In Spain, these species are cultured mainly in the Galician coast (NW Spain). In 2019 the local Galician production achieved 1917 tonnes with an economic value at first sale of 21.6 million Euros, of which the Japanese carpet shell corresponded 74% (12.1 million Euros), the Grooved carpet shell 10% (5.1 million Euros) and the slug carpet shell 16% (4.4 million Euros)^1–3,16,17^.

In these terms, clam production has become an interesting economic resource for European countries and Spain in particular. The natural populations have been decreasing during the last decades, consistently also have been the global captures^1–3^. The combined effect of several diseases (like those caused by parasites, virus and bacteria), natural hazards (such as competitors and predators) and increasing pollution of coastal areas, in addition to overfishing and/or improper management has severely affected the demographic growth of these mollusks.

All the aforementioned factors add up to the increase of demographic pressure^1–3,16^. This has forced the Spanish government to fund ambitious initiatives in collaboration with hatcheries on restocking clam populations without significant results^17–21^. The industry has begun to opt for hatchery spat production, by conditioning of adults for spawning and development of juveniles or catching spat from the wild but the variation of natural seed recruitment and irregular seed supply through the years has impeded aquaculture improvement and profitable inland production. In countries like China and Spain, significant economic costs are destined to protect ocean floors, where all competitors, seaweed and predators are persistently removed and the bottom smoothed to promote clam demographic growth^1–3,11,22^.

Cryopreservation has become a powerful tool for improvement of hatchery spat and overcome the limitations of aquaculture industry. Cryopreservation can provide a sustainable supply of competent shellfish juveniles irrespective of the season; hence it can avoid the reliance to wild catches. It has been really promising to ensure the implementation of selective breeding programs on aquaculture, enabling the possibility to make crosses of preserved families whose genes provide resistance to adverse events or certain diseases, which is also really encouraging for the restock of natural populations and decrease of fishing pressure on shellfisheries^23–27^. The cryopreservation knowledge focused on aquatic species is scarce and the cryopreservation protocols currently developed are focused on punctual species of high economical value like oysters^23,25,26,28–33^. Focusing on the current status of marine cryopreservation, the efficiency of the sperm protocols is consistent and widely extended but the unsuccessful attempts to cryopreserve mollusk oocytes compromise the storage of both parental genetic information at ultra-low temperatures^26,32,34–36^. Therefore, the present study has focused on larval stages following Heres et al. ^37^ and Rodriguez-Riveiro et al.^38^ guidelines. In general, research is focussed first on the short-term effects of cryopreservation to set the basis achieving maximal survival rates and larval fitness 48 h after thawing to scale the success long-term. Critical development points like gastrulation have to be overcome in order to obtain normal larvae after 48 h, therefore the presence of normal D-shaped larvae at 48 h is a strong indicator of a valid protocol^31,32,38–43^.

For the first time, cryopreservation research involves clam species, which play an important role on small-scale and local industry and their natural resources are declining. The main goal of this work is to provide a detailed investigation of cryopreservation procedures on the cited clams. The toxicity of common permeable CPAs, as well as different CPA combinations, increasing equilibrium times before slow-cooling and the post-thawing resilience of clam larval stages has been evaluated to further develop successful larval cryopreservation protocols for inland clam spat production enhancement.

## Results

### Toxicity tests

In general, the CPA addition altered the normal larval development. Abnormalities consisted of smaller shell size, abnormal shell shape and mantle protuberances on developed D-larvae. Moreover, disruption on development and larval mortality were observed, especially at high CPA concentrations.

**Figure 3:**
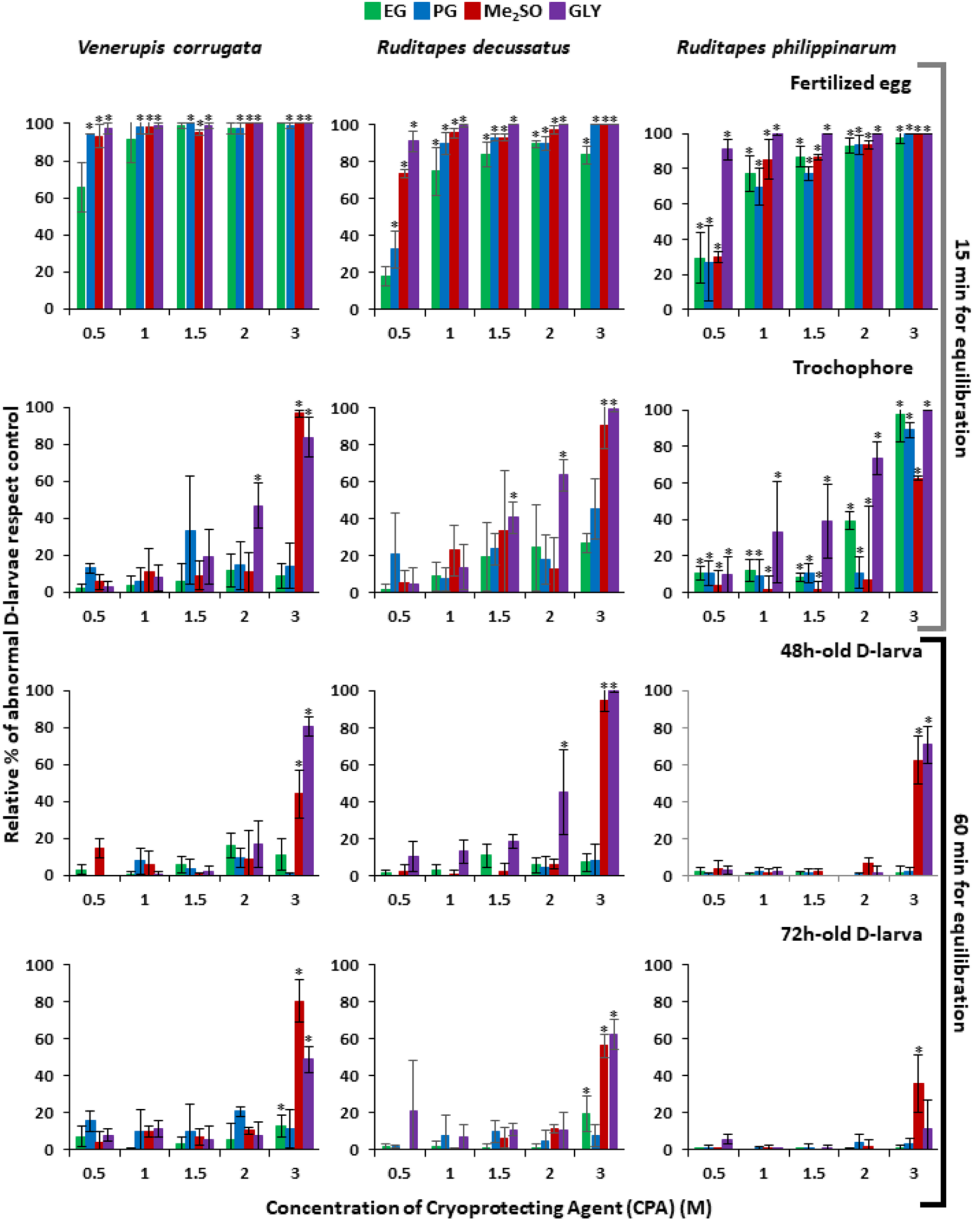
Percentage of abnormal D-larvae compared to control group developed from development stages of *Venerupis corrugata, Ruditapes decussatus* and *Ruditapes philippinarum* (fertilized egg, trochophore, 48 and 72 h-old D-larva) exposed to increasing concentrations of ethylene–glycol (EG), propylene-glycol (PG), dymethil-sulfoxide (Me_2_SO) and glycerol (GLY)*.* Results are expressed as mean ± standard deviation (SD), n = 3. Asterisk shows statistical differences with control assessments (p˂0.05). This parameter has been calculated as the division between the percentage of abnormal D-larvae calculated divided by the abnormality percentage showed in controls. Abnormality in controls was in general below 25% and the average percentage of abnormality was 16 ± 7.9.

The effect of CPA toxicity was similar among the three clams selected for this experiment: EG and PG resulted the least toxic CPAs, whereas Me_2_SO and GLY exposure were more detrimental, showing a dose–response relationship. The harmful effects were more notable depending on development stage, being more pronounced on the fertilized egg, where mortality rates were close to 100% over 1 M for all CPAs. Because of that, NOEC levels could not be determined as they were lower than the minimal concentration tested. On the other side, D-larva was the most resistant stage, thus there was not any significant effect at certain high CPA concentrations. Hence, the LOEC levels could not be calculated at the CPA concentrations tested in this experiment, they were lower than needed for such determination (Fig. 3, Table 1).

**Table 1:** NOEC (no observed effect concentration) and LOEC (lowest observed effect concentration) levels (mol/L) for development stages (fertilized egg, trochophore, 48 and 72 h-old D-larva) of *Venerupis corrugata, Ruditapes decussatus* and *Ruditapes philippinarum* after exposure to increasing concentrations of the following cryoprotecting agents (CPAs): ethylene–glycol (EG), propylene–glycol (PG), dymethil-sulfoxide (Me_2_SO) and glycerol (GLY)*.*

	Fertilized egg		Trochophore		48 h-old D-larva		72 h-old D-larva	
	NOEC (M)	LOEC (M)	NOEC (M)	LOEC (M)	NOEC (M)	LOEC (M)	NOEC (M)	LOEC (M)
***Venerupis corrugata***								
Me2SO	–	0.5	2	3	2	3	2	3
GLI	–	0.5	1.5	2	2	3	2	3
EG	3	–	3	–	3	–	2	3
PG	–	0.5	3	–	3	–	3	–
***Ruditapes decussatus***								
Me2SO	–	0.5	2	3	2	3	2	3
GLI	–	0.5	1	1.5	1.5	2	2	3
EG	0.5	1	3	–	3	–	2	3
PG	-	0.5	2	3	3	–	3	–
***Ruditapes philipinarum***								
Me2SO	–	0.5	1.5	2	2	3	2	3
GLI	–	0.5	0.5	1	2	3	3	–
EG	–	0.5	2	3	3	–	3	–
PG	0.5	1	1.5	2	3	–	3	–

Regarding to toxicity results, EG and PG were selected as CPA solutions for further cryopreservation experiments, in order to determine which one had the higher cryoprotection effect for clam larvae.

### Cryopreservation experiments

**Figure 4:**
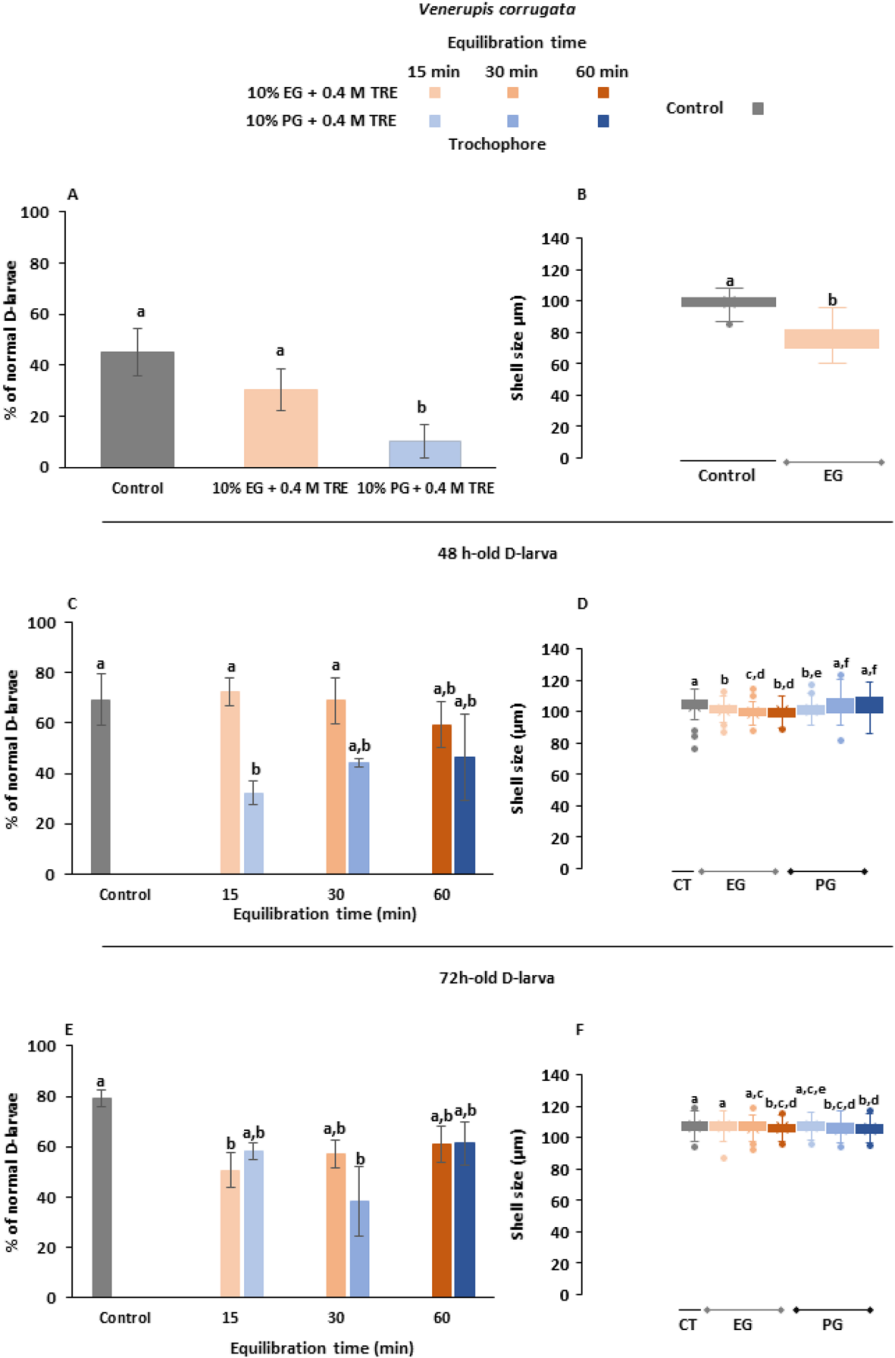
Post-thaw fitness parameters on larvae of *Venerupis corrugata* cryopreserved at trochophores, 48 and 72 h-old D-larval stages: **(A)** survival of cryopreserved trochophores; **(B)** shell size of developed D-larvae from cryopreserved trochophores; **(C)** survival of cryopreserved 48 h-old D-larvae; **(D)** shell size of developed D-larvae from cryopreserved 48 h-old D-larvae; **(E)** survival of cryopreserved 72 h-old D-larvae; **(D)** shell size of developed D-larvae from cryopreserved 72 h-old D-larvae. Larvae were cryopreserved using two CPA solutions: 10% ethylene–glycol (EG) + 0.4 M trehalose (TRE) and 10% prophylene-glycol (PG) + 0.4 M TRE (final concentrations), both prepared in filtered sea water (FSW). Increasing equilibrium times were tested on D-larvae. Results are expressed as Mean normal D-larval percentage ± Standard Deviation, SD (n = 100, 3 replicates per treatment) or as box plots for shell size assessments (µm) considering mean value (Mean ± Standard Deviation, SD) (n = 35, 3 replicates per treatment). Different letters show statistical differences with p˂0.05.

The success of *V. corrugata* larval cryopreservation is represented in Fig. 4, considering post-thawing survivorship and shell size parameters. Focusing on survival from trochophore cryopreservation, the use of 10% EG + 0.4 M TRE yielded 30 ± 8.39% of normal D-larvae, significantly better than the CPA solution containing PG as permeable CPA (10 ± 6.56%) (Fig. 4A). Overall, there were significant differences between the control group and treatments, which had 45 ± 9.17% of larval recovery. Shell lengths were not measured for the treatment with PG due to the lack of normal larvae. Cryopreservation procedure yielded a significant delay (22.81%) on larval growth; whereas control larvae achieved a mean value of 99 ± 4.44 µm, cryopreserved larvae with 10% EG + 0.4 M TRE had 76 ± 8.43 µm (p < 0.05) (Fig. 4B).

Cryopreservation of older development stages produced better results: in the case of 48 h-old D-larva, the survivorship was on samples cryopreserved with EG (Fig. 4C). Control group recovered 69 ± 10.02% 48 h after thawing, less than 72 ± 5.69% of normal larvae showed in 10% EG ± 0.4 M TRE allowing 15 min for equilibration, which was the best combination. The increase of the equilibrium time seemed to be detrimental when using EG, hence survival of cryopreserved samples exposed to CPA solution for 60 min descended to 59 ± 8.96%. However, statistical significance between these groups were not found (p > 0.05). In opposite, the survival descended with PG solutions but tended to be better when increasing exposure period for cryopreserved D-larvae. Significance was observed between 10% PG ± 0.4 M TRE, 15 min of equilibration and unfrozen larvae and 10% EG ± 0.4 M TRE, 15 or 30 min of equilibration (p < 0.05). Focusing on shell size (Fig. 4D), the differences among treatments were low although significance was achieved on samples exposed for the longest equilibration time tested with unfrozen larvae (p < 0.05). According with survivorship, there was a decrease on shell length of cryopreserved samples with EG, being more pronounced (3.62%) when the exposure time was 60 min. On the contrary, when using PG, the larval size was better with extended equilibrium period. The best value was obtained with 10% PG ± 0.4 M TRE and 60 min for equilibration (104 ± 6.55 µm), 0.7% higher than 103 ± 3.01 µm of control group.

Cryopreservation experiments on 72 h-old D-lava yielded significant differences between unfrozen larvae and 10% EG + 0.4 M TRE, 15 min for equilibration and 10% PG + 0.4 M TRE, 30 min for equilibration (p < 0.05), but there was not found any statistical significance when comparing treatments (p < 0.05). The highest survival percentage was over 61%, obtained with 10% EG + 0.4 M TRE and 10% PG + 0.4 M TRE, 60 min for equilibration (Fig. 4E). A progressive descend on shell length was hardly appreciated by cryopreservation, although significance was found in samples using equilibrium times of 30 and 60 min and sightly pronounced on PG treatments (from 0.41 to 1.36%). Only the larvae exposed for 15 min did not show significant differences with control group, which values were close to 107 µm with a difference of 0.03% (p > 0.05) (Fig. 4F).

**Figure 5:**
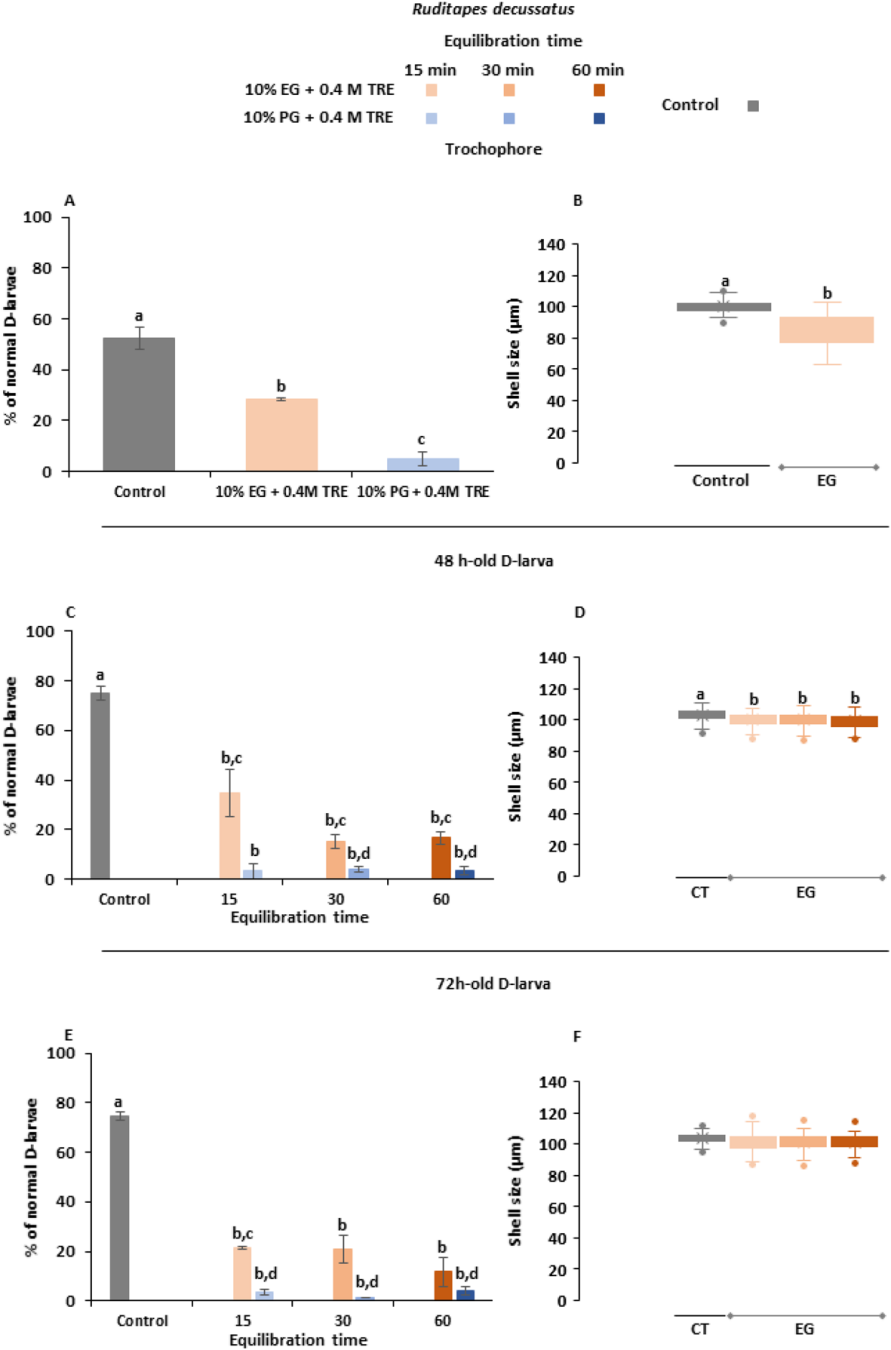
Post-thaw fitness parameters on larvae of *Ruditapes decussatus* cryopreserved at trochophores, 48 and 72 h-old D-larval stages: **(A)** survival of cryopreserved trochophores; **(B)** shell size of developed D-larvae from cryopreserved trochophores; **(C)** survival of cryopreserved 48 h-old D-larvae; **(D)** shell size of developed D-larvae from cryopreserved 48 h-old D-larvae; **(E)** survival of cryopreserved 72 h-old D-larvae; **(D)** shell size of developed D-larvae from cryopreserved 72 h-old D-larvae. Larvae were cryopreserved using two CPA solutions: 10% ethylene–glycol (EG) + 0.4 M trehalose (TRE) and 10% prophylene–glycol (PG) + 0.4 M TRE (final concentrations), both prepared in filtered sea water (FSW). Increasing equilibrium times were tested on D-larvae. Results are expressed as mean normal D-larval percentage ± standard deviation, SD (n = 100, 3 replicates per treatment) or as box plots for shell size assessments (µm) considering mean value (Mean ± Standard Deviation, SD) (n = 35, 3 replicates per treatment). Different letters show statistical differences with p˂0.05.

Focusing on results from experiments on *R. decussatus* larvae (Fig. 5), the percentage of normal D-larvae differed significantly respect control groups (p < 0.05). For all development stages, CPA solution containing 10% EG + 0.4 M TRE yielded higher post-thawing survival rates than selecting PG as permeable CPA. 28 ± 0.58% of trochophores metamorphosed into D-larva cryopreserved with EG, lower than recovery of control group (52 ± 4.51%). When using PG, survivability fell to 5 ± 2.65% (Fig. 5A). In addition, shell size was significantly delayed (15.84%) compared to control group (Fig. 5B), where mean of cryopreserved larvae was 84 ± 9.18 µm and mean of unfrozen larvae was 100 ± 3.92 µm (p < 0.05).

In the case of D-larvae, survival of control groups at day 48 post-thawing were significantly higher, close to 75% (p < 0.05). For both D stages, the increase of equilibrium time seemed to be detrimental. Focusing on 48 h-old D-larval cryopreservation, the highest percentage of normal larvae was achieved with 10% EG + 0.4 M TRE and 15 min for equilibration, obtaining 34 ± 9.29% (Fig. 5C). Regarding larval growth, shell length assessments gave a mean of 103 ± 3.66 µm for unfrozen larvae, which was over 3.37% significantly higher than cryopreserved trials. There were not significant differences between treatments. Larvae cryopreserved in 10% EG + 0.4 M TRE and 15 min for equilibration provided highest shell lengths (99 ± 3.7 µm) (Fig. 5D).

According with results of 48 h-old D-larva, the oldest larval stage showed the best recovery when cryopreservation was carried out using 10% EG + 0.4 M TRE allowing 15 min for equilibration (21 ± 0.58%), achieving significant differences with larvae cryopreserved in CPA combinations containing PG (p < 0.05) (Fig. 5E). Shell lengths did not reveal significant differences between treatments (p > 0.05), but the control group achieved the highest mean size (103 ± 3.35 µm) followed by 10% EG + 0.4 M TRE, 60 min of equilibration, which reached a mean 2.32% lower than control, 101 ± 4.43 µm (Fig. 5F).

**Figure 6:**
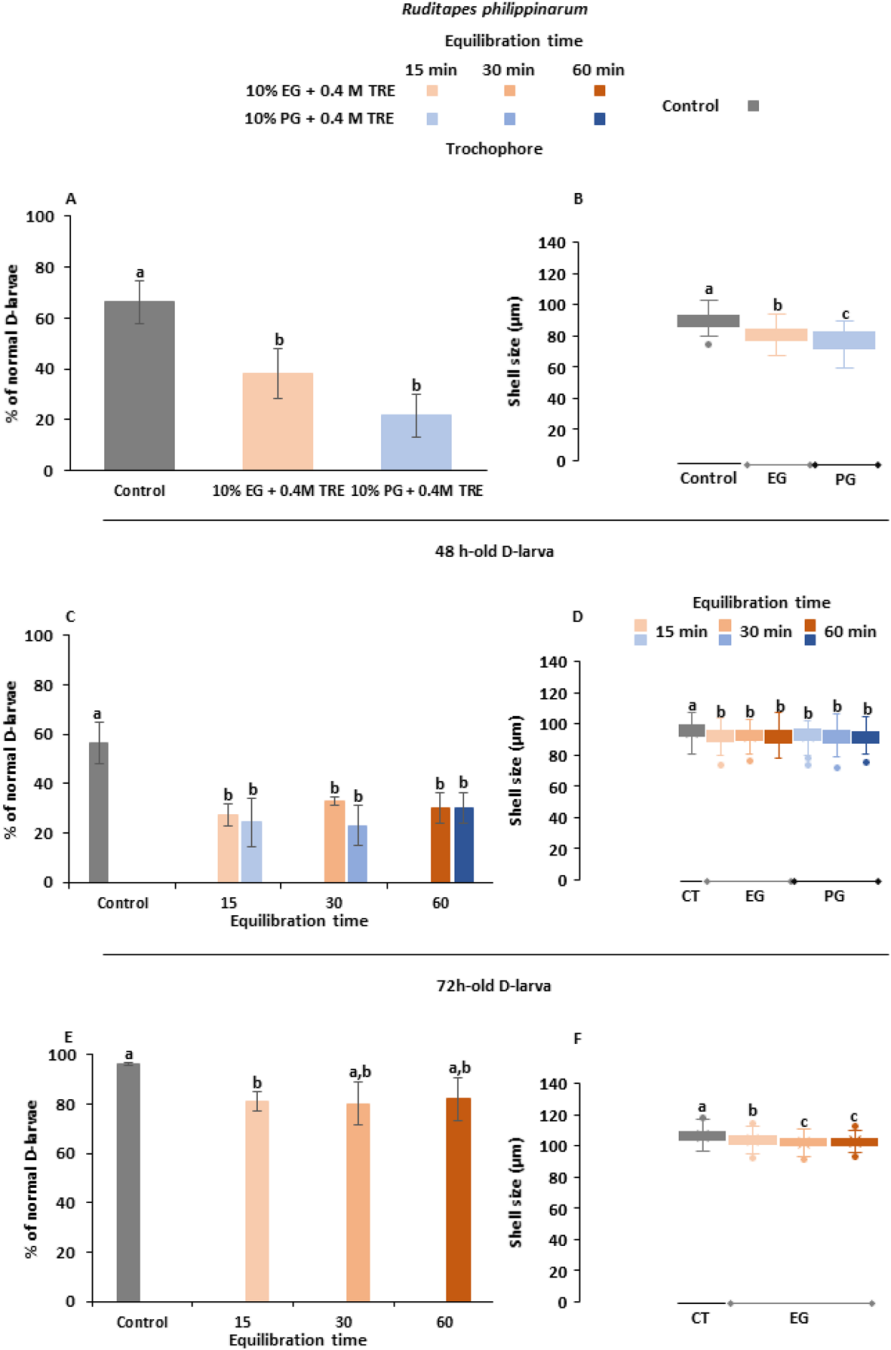
Post-thaw fitness parameters on larvae of *Ruditapes philippinarum* cryopreserved at trochophore, 48 and 72 h-old D-larval stages: **(A)** survival of cryopreserved trochophores; **(B)** shell size of developed D-larvae from cryopreserved trochophores; **(C)** survival of cryopreserved 48 h-old D-larvae; **(D)** shell size of developed D-larvae from cryopreserved 48 h-old D-larvae; **(E)** survival of cryopreserved 72 h-old D-larvae; **(D)** shell size of developed D-larvae from cryopreserved 72 h-old D-larvae. Larvae were cryopreserved using two CPA solutions: 10% ethylene–glycol (EG) + 0.4 M trehalose (TRE) and 10% prophylene-glycol (PG) + 0.4 M TRE (final concentrations), both prepared in filtered sea water (FSW). Increasing equilibrium times were tested on D-larvae. Results are expressed as mean normal D-larval percentage ± standard deviation, SD (n = 100, 3 replicates per treatment) or as box plots for shell size assessments (µm) considering mean value (mean ± standard deviation, SD) (n = 35, 3 replicates per treatment). Different letters show statistical differences with p˂0.05.

The overall survival rate observed after cryopreservation experiments on *Ruditapes philippinarum* individuals showed a significant difference between control and cryopreserved groups at all of the development stages selected (p < 0.05), excepting when 72 h-old D-larvae were cryopreserved with 10% EG + 0.4 M TRE allowing 30 and 60 min for equilibration (p > 0.05). Trochophore cryopreservation was significantly higher with EG (38 ± 9.71%) than using PG (21 ± 8.39%) (Fig. 6A). In agreement with survival results, control group reached the biggest mean value, 89 ± 5.44 µm, whereas larvae cooled in 10% EG + 0.4 M TRE were significantly bigger (80 ± 5.51 µm) than larvae cryopreserved in 10% PG + 0.4 M TRE (76 ± 7.23 µm) (p < 0.05) (Fig. 6B).

48 h old-D larva cryopreserved with 10% EG + 0.4 M TRE and 30 for exposure to CPAs before slow-cooling yielded the highest survival value: 33 ± 1.73%. In this case, significant differences between treatments were not found (p > 0.05) (Fig. 6C). Statistical analysis of shell size showed significant differences between unfrozen larvae and cryopreserved ones (p < 0.05), but not between treatments (p > 0.05). Unfrozen larvae reached a mean value of 95 ± 5.44 µm, being the larvae cryopreserved with 10% EG + 0.4 M TRE and 30 in of equilibration the biggest ones, with a mean of 92 ± 5.03 µm, 2.92% lower (Fig. 6D).

Recovery of 72 h-old D-larva was high and results were similar among trials (Fig. 6E). Control group had 96 ± 0.58% of survivorship, followed by the treatment 10% EG + 0.4 M TRE and 60 min of equilibration with 82 ± 8.72%, then 10% EG + 0.4 M TRE and 15 min of equilibration (81 ± 4%) and finally 10% EG + 0.4 M TRE and 30 min of equilibration (80 ± 8.66%). Results using CPA solutions containing PG were unsuccessful. According to other experiments, cryopreservation yielded an average delay of 3.59% on larval growth but without significance (p > 0.05). Larvae of control group averaged 106 ± 3.89 µm, followed by the treatment 10% EG + 0.4 M TRE, 15 min for equilibration (103 ± 3.47 µm) (Fig. 6F).

**Figure 7:**
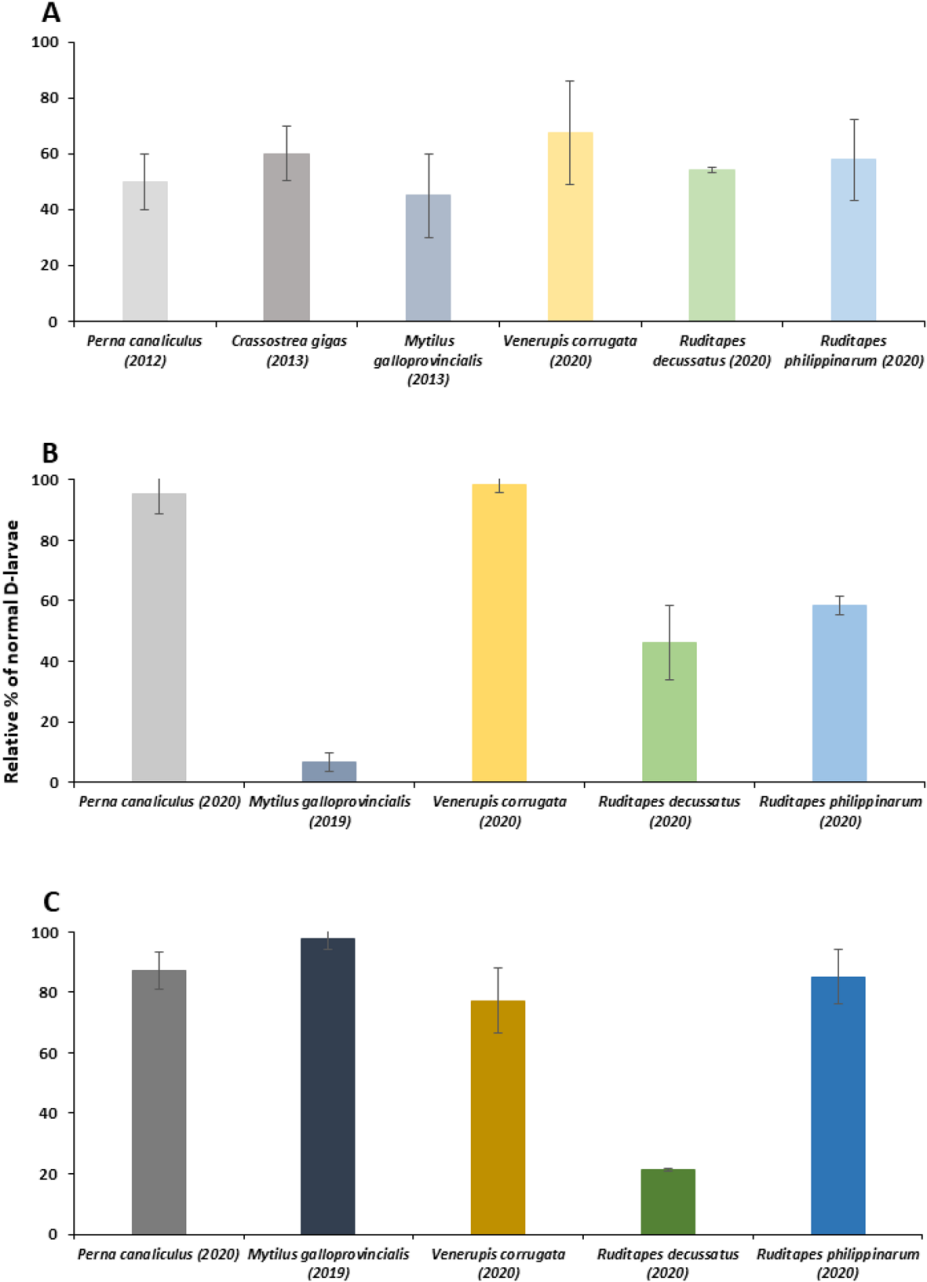
Percentage of normal D-larvae developed from cryopreserved trochophores **(A)**, 48 h- old D-larvae **(B)** and 72 h-old D-larvae **(C)** compared to control group of related marine mollusks (mean ± SD), data from^31,32,37,44^. The color of bars represents each species, and the tone gets increasingly intense alongside development.

Figure 7 compares the success of cryopreservation protocols developed for related mollusk species^31,32,37,38^ assessed as relative percentage of normal D-larvae developed from cryopreserved individuals compared to control groups. Regarding cryopreservation of trochophores (Fig. 7A), relative survival rates were comprised between 40 and 70%, of which the slug carpet shell cryopreservation corresponded the highest result, 67 ± 18.64%. In the case of D-larvae, results differ more between species. 48 h-old D-larval cryopreservation of the slug carpet shell obtained the highest larval recovery, followed by the cryopreservation protocol described for 48 h-old D-larva of the Greenshell mussel™ (*Perna canaliculus*), close to 100%^44^ (Fig. 7B). Cryopreservation of 72 h-old D-larva reported better results and in most of the cases, so the relative survival was superior to 80%, excepting cryopreservation of *R. decussatus* (Fig. 7C).

## Discussion

The preservation of genetic material by cryopreservation procedures provides several advantages for the restock endangered species wild populations, enabling long-term banking of genes from endangered/overfished species avoiding the total loss of genetic information which could be useful for further research on repopulation. This conservation effort, with the use of cryopreservation and biobanking of genetic material is widely extended on threatened terrestrial groups. Several reports have been published describing cryopreservation or vitrification protocols for endangered species, both animals and plants^45^. Published protocols are developed for a wide variety of cell-types depending on the species, including somatic cells, gonad tissues, embryos or gametes. Regarding aquatic organism conservation, the research is mostly centred on coral reefs^46,47^ due to their ecological importance and their high risk of diversity loss. Sperm cryopreservation is also widespread on other organisms, mostly commercial species and threatened species^48,49^. On the contrary, oocyte cryopreservation is a complicated endeavour in aquatic organisms. The alternative, when cryopreserving eggs is impossible, is the cryopreservation of embryos and larval stages to achieve the long-term storage of the total genetic information of aquatic organisms. This is the case of mussels or oysters and clams, where eggs are not possible to cryopreserve so far, there have been several protocols working toward the development of successful protocols to preserve larval stages.

Supporting the usefulness of the selection of this development stage, there are published studies in which the development of larval cryopreservation protocols has been proven very successful on mollusks of high economical value. The effort of these protocols consists on the improvement of aquaculture production and to produce mollusk spat inland in efficient ways^23,26,44,50–52^. This is the first time three related clam species, whose natural populations are declining, have been selected for servation experiments to develop robust and reproducible cryopreservation protocols as successful approach for their conservation long-term.

Results suggest that EG should be chosen for clam larval cryopreservation according with its toxicity and cryoprotection benefits during the cryopreservation process, this agrees with other publications with molluscs such as^37,53,54^ suggesting that Molluscs from the order Bivalvia respond better to higher ethylene glycol concentrations thus allowing for better cryoprotection during freezing whereas they are very sensitive to Me_2_SO toxicity. Other reports where other CPAs were used, such as Me_2_SO, obtained lower post-thawing survival rates, whose mortality could be due to the detrimental effect of them on larvae, as reported in the present work^42,55^.

The first step for the development of a cryopreservation protocol, from our point of view, consists of the determination of the best CPA attending to toxicity, in order to avoid the added harmful effect that leads abnormalities or lethal effects by selecting an improper one^26^. The toxicity of CPAs can be due to a combination of two factors: the osmotic shock at CPA addition step and/or interactions with intracellular molecules which lead to disorders on cell processes. For example, the high harmful effect of GLY may be given because of its high molecular weight and consequent low permeability which yields detrimental cell volume changes^56^. In the case of Me_2_SO, it is known its capacity to inhibit peroxide enzymes^57–59^. Because of that, it is important to take into account the method chosen for addition step and equilibration time, beside type of CPA and concentration.

Toxicity tests shown here revealed EG and PG were generally the least toxic CPAs for the three species, whereas the exposure to Me_2_SO and GLY resulted damaging, especially at high concentrations. This agrees with conclusions from toxicity tests on development stages of the bivalve *Mytilus galloprovincialis* and related mollusks^37,53,54^, where there was also a similar response throughout development stages and toxic effect of CPAs. The high sensitivity of eggs and fertilized eggs led to high mortality rates, even from low CPA concentrations, probably one of the main reasons why they have not been cryopreserved so far.

Results of toxicity tests were taken into account for the development of the final cryopreservation protocol, both EG and PG were chosen to study their capacity of cryoprotection, in combination with TRE. Trehalose is a quite popular non-permeable CPA whose benefits and its capacity to reduce toxicity of permeable CPA have been repeatedly reported in aquatic organisms^25,26,39,60–63^. In general, PG was worse cryoprotectant than EG. The present report shows the importance of study CPA toxicity, as well as CPA cryoprotection and optimize CPA concentrations considering the balance between them. EG has been selected for cryopreservation of development stages of related species before and it seems that this CPA could be the proper one for bivalve species^31,32,38,40,44,51,52,54^ in general.

Comparing between clam species, the highest survival rates using PG were obtained with *V. corrugata*. *R. decussatus* cryopreservation was really unsuccessful, but the use of EG improved post-thawing outcome although statistical significance was not showed between treatments. In contrast, the other clam specie from Genus *Ruditapes*, *R. philippinarum*, showed high tolerance to cryopreservation procedure. More research is needed to clarify the role of genetic divergence between these species on cryopreservation success since the results obtained here does not correspond with the phylogenetic relationship from^5^ (Fig. 2), where *V. corrugata* and *R. decussatus* are in the same clade group. It is well known the higher tolerance to adverse conditions of *R. philippinaru*m and this could make it more suitable for handling, cryopreservation and in vitro conditions than *R. decussatus*^64–66^.

Regarding which is the best larval stage, this study along several others on bivalves^32,37,38,44^ determines that D-larvae are more suited larval stages for cryopreservation protocol development, meanwhile trochophores despite being younger, not having yet a proto-shell and with simpler cellular organization, they are more fragile to handling, more sensitive to CPA toxicity despite of shorter exposure time and their cryopreservation has a lower success rate. D-larval stage is provided by a ciliated velum, perceptible stomach, and a prodisoconch I with D-shape^13^, which role on cryopreservation and permeability to CPAs is not clear and could play a key role on intrinsic tolerance to CPAs, hence the lower abnormality rates found after 60 min of exposure. The higher lipid content in trochophores destined as energy reserve for development could make them more vulnerable^40,67–69^. Light microscopic analyses^54,70^ showed minor cell-specific damages on both trochophore and D-larval stages after cryopreservation. These damages could lead to significant alterations on further development, specially at trochophore stage where neurogenesis stunt was observed. The degree of cell differentiation and associated cell membrane complexity could be responsible of the lower chilling trochophore tolerance and compromise larval development. Further investigation could consider lipid supplementation to enhance the resistance to chilling injury, successfully tested on related aquatic species^55,71,72^. Modifications on plasmatic membranes due to differentiation process can imply different permeating response to CPAs, thus research on terrestrial mammals has pointed out a general trend of increased permeability to CPAs when embryos develop^73–75^. This trend may be extended to other groups, including bivalves, hence CPA cryoprotection could be enhanced and widespread across all the animal cells enhancing post-thawing recovery at older larval stages. In the present study, the most tolerant development stage was the 72 h-old D-larva, although the harmful effect of Me_2_SO and GLY was still evident at high concentrations. This is supported by our work showing significant declining on survivorship from the lowest CPA concentrations in the case of fertilized eggs.

However, contrary to our findings, for other marine organisms such as corals, larval cryopreservation requires early-life stages because CPA permeability decreases with increasing larval age^76^. This is in agreement with traditional add-on approaches attempted to cryopreserve complex systems, like human tissues and organs, where their physical properties (diversity of cell types, cell densities, morphological differences and the interactions between cells and cell–matrix) become their cryopreservation really challenging. In addition, the specificity for each cell type, which has optimal cryoprotecting requirements, as well as cooling and warming rates, adds more problems to cryopreserve different cells at once^77–84^. Our work and prior research suggest that a balance between life-stage resilience and cell differentiation should be taken into account when choosing a development stage for cryopreservation.

This is the first time that research has considered three clam species from family Veneridae for a detailed study on larval cryopreservation. The comparative investigation reported here evidences a strong trend of which older bivalve development stages are more tolerant to CPA toxicity and cryopreservation procedures. Furthermore, EG seems to be the most recommended CPA for D-larval cryopreservation regarding its balance between low toxicity and high cryoprotection using 10% EG + 0.4 M TRE in sea water. Apparently, the equilibrium time is not a crucial step for clams as it seems to be for mussels, nonetheless authors suggest allowing maximal exposure to CPAs prior cryopreservation to ensure maximal cryoprotection (15 min for trochophores and 60 min for D-larvae). The cryopreservation protocol^27^ consists of cooling at 1 °C/min from 4 °C to − 12 °C, check for seeding if necessary, then cool down at 1 °C/min to − 35 °C and store in liquid nitrogen. These general conclusions could enable the establishment of standard guidelines for the development of further cryopreservation protocols of species within the same order, adapting the methodology in function of cell requirements in efficient ways. Further research should be focused on the capacity to produce competent clam spat from cryopreserved larvae and its ability to develop successfully. The improved cryopreservation protocols could significantly benefit inland spat production and help the aquaculture industry but also the species conservation effort by enhancing the restock of natural populations in declining.

## Materials and methods

### Gamete collection and handling

Mature clams (*Venerupis corrugata* (Gmelin, 1971), *Ruditapes decussatus* (Linnaeus, 1758) and *Ruditapes philippinarum* (Adams & Reeve, 1850)) were obtained from the wild in the south margin of Ria de Vigo (Galicia, NW Spain) and deposited in PVC tanks with Filtered Sea Water (FSW, 35–37‰, filtered through 0.22 μm mesh and irradiated with Ultraviolet radiation to avoid the presence of microorganisms). Water temperature was changed from 14 to 20 °C to induce the clam spawning. Spawning individuals were put into separate plastic containers to collect sperm and oocytes. Oocyte quality and maturity were examined focusing on their shape and colour before fertilization, selecting those females that produced eggs with homogenous oval shape and high content of lipid droplets. Sperm was checked for motility under microscope^41,43,61^ (DiMatteo et al., 2009). Gametes from a pool of males and a pool of females were collected and transferred into FSW separately, in order to minimize genetic variability^85,86^. A small volume of sperm was added to the oocyte suspension (approximately a sperm:oocyte ratio of 20:1) and a 15-min contact period was allowed before evaluation of the percentage of fertilization by counting oocytes with formation of the first polar body. A portion of fertilized eggs were retrieved for toxicity tests. The resting embryos were incubated at 18–20 °C in a density of 40 individuals/mL. They were periodically sampled by draining the incubation tank through a 63 µm screen to collect, trochophore larval stage (18–20 h post-fertilization) and 48 and 72 h-old D-larval stage individuals for experiments. In all experiments, the endpoint was the number of normal/abnormal larvae obtained 48 h post-cryopreservation, in the case of the earlier stages a high presence of normal D-shaped larvae at 48 h post-thaw is a strong indicator of a valid protocol^31,32,38–43^. In the case of larvae already cryopreserved at the D-stage the time allowed for post-thaw incubation was also 48 h for consistency.

### Cryoprotecting reagents

All chemicals were purchased from Sigma Aldrich chemicals (St Louis, MO, USA). Cryoprotecting agent (CPA) solutions were prepared at twice the final concentration for the experiments. Therefore, when the same volume of stock solution and larvae suspension were mixed, the required final chemical concentration was produced (added in proportion 1:1). For toxicity tests, increasing concentrations of Ethylene–Glycol (EG), Propylene-Glycol (PG), Dimethyl-Sulfoxide (Me_2_SO) and Glycerol (GLY) were prepared in FSW. For cryopreservation experiments, two CPA solutions a) 10% (v/v) EG + 0.4 M (w/v) trehalose (TRE) and b) 10% (v/v) PG + 0.4 M (w/v) TRE (final concentrations) were selected according to conclusions from toxicity tests and results of^32,37^.

### Toxicity tests

In order to select the proper permeable CPA for each clam species and development stage, increasing concentrations of the most used permeable CPAs on marine cryopreservation^37^, such as PG, Me_2_SO, EG and GLY were selected. Concentrations ranged from 0.5 to 3 M (final concentrations). A sample of larvae was retrieved and put into 20 mL FSW for further incubation and perform control groups. Three replicates were assayed for each CPA concentration and control trials. Cryoprotectant solutions were added to a FSW mL with clam individuals in one step (n = 3, 400–600 individuals per replicate). Prior research on marine invertebrates pointed out that the addition of CPAs in a single step is not lethal for marine mollusk development stages^31,32^). However, it should be mentioned that for other marine invertebrate species, such as sea urchin, it is required the addition of CPA solutions following a gradual stepwise protocol to reduce toxicity and osmotic stress, which could lead to cell damage^62^. 15 min were allowed to reach equilibration when fertilized egg and trochophore larva were used at room temperature (18–20 °C). This period was extended to 60 min for D-larval stages. Actually, there are no mathematical calculations for CPA permeation in clam individuals, but empirical data (as permeability parameters of the CPAs have not been calculated) focused on other bivalve species seems to indicate that 15 min-exposure is time enough to reach osmotic equilibration in the case of earlier development stages^31,32^. However, previous work in our lab demonstrated the beneficial effects on post-thawing larval survival and fitness of increasing equilibrium time from the recommended 15 min to 60 in the case of the D-larva^32,37^. After exposure and equilibration, samples were diluted with FSW at 1:1, allowing 2 min for recovery at room temperature (18 ± 1 °C). Then, samples were filtered using a 40 µm membrane filter and rinsed with abundant FSW to remove the CPA. They were incubated in 20 mL of clean FSW at 18–20 °C at a density of 20–30 larvae/mL until D-larval stage was reached for exposed fertilized eggs and trochophores; allowing 48 h of incubation in the case of D-larval stage. After that, cells were fixed with formalin for larval normal development assessment.

### Cryopreservation trials

Cryoprotecting solutions were added to a FSW mL with 400–600 clam individuals. It took 15 min to reach equilibration for trochophores (18–20 °C). When cryopreserving the D-larvae, three increasing equilibrium times (15, 30 and 60 min) were tested additionally. The samples were loaded into 0.25 mL straws after CPA addition, sealed with PVC powder (IMV Technologies, France) and placed in a Freeze Control System (Cryologic, Pty Ltd, Mt Waverley, Australia). For each experiment, control group consisted of larvae diluted in FSW loaded in straws at the same density than cryopreserved trials and deposited in 20 mL FSW for incubation. Three replicates were assayed for each treatment and control trials. They were cryopreserved following cryopreservation protocol for larvae of *M. galloprovincialis* (Paredes et al., 2021) as described: holding at 4 °C for 2 min, then cooling at 1 °C/min to − 12 °C, holding at − 12 °C for 2 min, cooling 1 °C/min to − 35 °C, plunging into liquid nitrogen. Larvae were thawed by immersion into a water bath at 35 °C for 6 s. Seeding at − 12 °C was carried out manually if needed by dipping forceps into LN2 and then touching the top of each straw in the freezer with the lid removed^31^. Then, thawed samples were diluted 1:1 with FSW and samples were filtered through a 63 µm mesh to remove the CPA and incubated into clean FSW at 18–20 °C until D-larval stage was reach in the case of cryopreserved trochophores; during 48 h for the rest of D-larval stages. Finally, samples were fixed in formalin for % normal D-larvae count.

### Larval abnormality criteria

The discrimination between normal D-larvae and abnormal D-larvae was determined under microscope attending to previous work focused on shell larval morphology and guidelines from other experts in the shell abnormalities and abnormally developing larvae of related mollusc in ecotoxicological larval bioassays^32,37,38,54,87,88^. Typical larval abnormalities found ranged from: delayed development (trochophores), deviations from the D-larvae shell shape like indented margins or hinge deformations (concave or convex hinges) or presence of clear protruding mantle.

### Statistical analysis

Data is expressed as mean ± Standard Deviation (SD). Statistical analyses were conducted using the IBM SPSS 15.0 version^89^ statistical software and according to^90,91^ when required. First, the percentage data was arcsine-transformed to achieve normality^92^. Normality distribution of transformed data was tested using the Shapiro–Wilk test (p > 0.05). Homogeneity of variances were checked using Levene’s test (p > 0.05). For toxicity tests, differences in the percentages of abnormal larvae among treatments were analysed by one-way of variance (ANOVA) followed by the Dunnett's test to calculate the NOEC (No Observed Effect Concentration) and LOEC (Lowest Observed Effect Concentration) levels. Post-thawing survival percentage was evaluated by one-way ANOVA, followed by multiple pairwise comparison using Bonferroni’s post-hoc test under the assumption of homogeneity of variances. When homogeneity of variances was not assumed, Dunnett T3 post-hoc test was selected for multiple pairwise comparison. Shell length data was analysed using non-parametric Mann–Whitney’s U test (p > 0.05). A p-value less than 5% was considered as significant.
